# PAR for the course – protease-activated receptor 4 protects the brain from hemorrhage

**DOI:** 10.1016/j.rpth.2025.103337

**Published:** 2026-01-02

**Authors:** Izabella Andrianova, Frederik Denorme

**Affiliations:** 1 Department of Emergency Medicine, Washington University School of Medicine, St. Louis, Missouri, USA; 2 Department of Veterinary Sciences, University of Antwerp, Antwerp, Belgium

In this issue of Research and Practice in Thrombosis and Haemostasis, Link et al. [1] show that protease-activated receptor (PAR) 4 deficiency significantly worsens traumatic brain injury (TBI) in mice. Using a controlled cortical impact model, the authors report that PAR4-deficient (PAR4^-/-^) mice experience higher early mortality, more extensive intracranial bleeding, and marked blood-brain barrier impairment compared with wild-type controls. These findings carry important implications. PAR4 inhibition has recently gained attention as a potentially safer antithrombotic strategy, including for ischemic stroke. The current study reminds us that PAR4 also supports hemostasis and vascular stability in the brain and that its loss can unmask a bleeding-prone state. The investigators used a severe TBI model that places extraordinary stress on the cerebrovascular system. Whether similar effects appear in more subtle or diffuse injury models remains an open and important question. As PAR4-targeted therapies move toward clinical translation, these results underscore the need for careful evaluation of the bleeding risk of PAR4-targeting strategies, particularly in the context of neurovascular injury.

PAR4 is 1 of 4 known PARs that are activated by thrombin. Thrombin activates PARs by enzymatic cleavage of the extracellular tail of the receptor [2], thereby generating a tethered ligand [3]. This tethered ligand subsequently binds the receptor, leading to the activation of associated G-proteins, resulting in a signaling event and the activation of the target cell. Human platelets express 2 of the 4 known PARs: PAR1 and PAR4. Both PAR1 and PAR4 have distinct signaling functions, leading to different dynamics of platelet activation [2]. While PAR1 activation leads to instant, unstable platelet activation, PAR4-induced activation is slower but prolonged and more sustained [3]. In human platelets, PAR1 is considered the main responsible receptor for thrombin-induced platelet activation. Therefore, PAR1 has become one of the main antithrombotic targets of antiplatelet therapy in preventing thrombin-induced platelet hyperactivation. Although PAR1 inhibitors are effective in preventing thrombosis, they are also associated with an increased bleeding risk, which is most pronounced in the brain [4,5]. This clinical experience underscores the need to carefully consider how modulating thrombin signaling pathways may influence neurovascular stability, particularly in individuals at risk of brain injury or hemorrhagic complications. This unfavorable side effect of PAR1 inhibitors has, in fact, made them become contraindicated for ischemic stroke patients and has pushed researchers to look for alternative targets as antiplatelet agents. One attractive alternative target is PAR4.

In recent years, several studies have shown that PAR4^-/-^ mice are protected in several models of thrombosis and immunothrombosis, including ischemic stroke [6], myocardial infarction [7], atherosclerosis [8], stenosis-induced venous thrombosis [9], and influenza [10]. However, preclinical *in vivo* research on PAR4 inhibition is challenging due to crucial differences between murine and human platelets. Relevant to this context, murine platelets lack PAR1, the key thrombin receptor in humans. Instead, murine platelets express PAR3 [3], which acts as a signaling adaptor [11], mainly facilitating the interaction between thrombin and PAR4. Therefore, PAR4 acts as the only functional signaling receptor on mouse platelets. As a result, it is important to keep in mind that the relative contribution of PAR4 to hemostasis and thrombosis is likely greater in mice than in humans. This also raises the possibility that complete PAR4 deficiency, as modeled in knockout mice, may overestimate thromboprotection and bleeding risks in humans, who will most likely be receiving only partial pharmacologic inhibition, with active PAR1 serving as a backup.

Taking these points into account, the current study [1] provides clear evidence for the role of PAR4 in maintaining vascular integrity in the brain, at least in mice. Interestingly, and counterintuitively, this observation was not made in ischemic stroke studies using PAR4^-/-^ mice or pharmacological inhibitors of PAR4. This discrepancy likely reflects the dual role of platelets in the injured brain. In ischemic stroke, platelets drive acute brain injury through inflammation and microthrombosis. Yet platelets also play a protective role in ischemic stroke brain by helping prevent hemorrhagic transformation caused by the vascular injury they initially contribute to. This duality is illustrated by studies of platelet depletion. Preventive platelet depletion reduces ischemic brain injury without increasing hemorrhagic transformation [12]. In contrast, platelet depletion 2 hours after stroke onset does not reduce injury but instead allows for hemorrhagic transformation [13], underscoring the timing-dependent roles of platelets in the ischemic brain. In this context, PAR4 deficiency or early pharmacologic inhibition likely prevents initial ischemic injury and therefore reduces the brain’s susceptibility to hemorrhagic conversion. An important next step will be to determine whether delayed PAR4 inhibition carries a similar bleeding risk in murine stroke models, paralleling what has been observed with delayed platelet depletion. The current study by Link et al. [1] suggests this might be the case.

Mechanistically, the authors did not observe evidence of enhanced neuroinflammation; however, this conclusion is based solely on gene expression analyses. Future studies should therefore incorporate more granular assessments of brain inflammation, including quantitative profiling of leukocyte recruitment and microglial activation. Given that PAR4 is expressed in neuronal tissue, it is plausible that its absence influences TBI outcomes beyond the acute time window examined here. Neuronal PAR4 may modulate processes such as synaptic function, cellular stress responses, and neurovascular integrity during the subacute and chronic phases of injury. In this context, the baseline neurological differences observed between the mouse strains become particularly relevant. These strain-dependent differences suggest that PAR4 may contribute to normal neurological function, and its deficiency could alter recovery trajectories after TBI in ways not captured by the acute assessments performed in the current study. Moreover, exploring additional cellular sources of PAR4 may also be informative in the acute phase, particularly since the authors report no differences in the number of recruited platelets. Platelet recruitment is only the first step in hemostasis, and reduced downstream platelet activation could contribute to increased hemorrhage. However, these findings also raise the possibility that PAR4 has functions outside of platelets that may influence acute TBI outcomes. Besides PAR4 on neuronal cells, PAR4 on endothelial cells may regulate vascular stability and potentially explain the increased blood-brain barrier leakage and immunoglobulin G extravasation observed in the current study.

Whether the marked effects of PAR4 deficiency on TBI outcomes in mice translate into a bleeding risk for humans treated with PAR4-targeted drugs remains uncertain and warrants further investigation. Finally, in addition to the translational hurdles ahead, it is important to note that the comparisons in this manuscript are between 2 groups of mice from 2 distinct colonies. This could have led to genetic drift in the background of these mice, complicating comparisons and weakening the causal role of PAR4 in the observed phenotype. Future studies should focus on using either littermate PAR4^+/+^ and PAR4^-/-^ mice or wild-type littermates randomized to receive either vehicle or a PAR4 antagonist.
